# Mining for crypto protection: a search for *Cryptosporidium* antibodies reveals antigens associated with immunity

**DOI:** 10.1172/JCI171966

**Published:** 2023-08-15

**Authors:** Ian S. Cohn, Christopher A. Hunter

**Affiliations:** Department of Pathobiology, School of Veterinary Medicine, University of Pennsylvania, Philadelphia, Pennsylvania, USA.

## Abstract

Infectious diarrhea is a major cause of morbidity and mortality, particularly for children in low- and middle-income countries. *Cryptosporidium* is a diarrheal pathogen for which there is no vaccine and current therapies are only partially effective. In this issue of the *JCI*, Gilchrist, Campo, and colleagues surveyed a large cohort of Bangladeshi children to profile antibody responses against an array of *Cryptosporidium* proteins. They discovered 233 proteins to which children developed antibodies, identified seven as being associated with protection from reinfection, and provided insights regarding the longevity of *Cryptosporidium* antibodies and the development of antibody breadth. In this commentary, we discuss the burden of disease caused by *Cryptosporidium* and how these studies highlight the strategies to better manage this parasite.

## The global burden of diarrheal diseases and *Cryptosporidium*

Diarrheal diseases are major causes of morbidity and mortality for children worldwide, particularly in low- and middle-income countries (LMICs). This burden is felt most strongly in children less than five years of age, as in this age group roughly one in nine deaths results from diarrheal disease (1). Repeated encounters with diarrheal pathogens in early childhood can lead to developmental impairment, chronic malnutrition, and stunting (2). Because the burden of these conditions is felt most strongly in LMICs, the global health community has undertaken efforts to develop more prevention and treatment strategies for infectious diarrhea (3, 4). One keystone of these efforts is the development of vaccines, exemplified by the success of the rotavirus vaccine in diminishing morbidity and mortality associated with this infection in pediatric patients (5–7). A seminal epidemiological study in 2013 involving 22,500 children under 5 years old from Africa and Asia highlighted the diversity of pathogens that cause severe diarrhea (2). Surprisingly, the protozoan parasite *Cryptosporidium* emerged second only to rotavirus as a cause of moderate-to-severe diarrhea in children under one year old and continued to be a risk factor in older age groups. While vaccines are available for rotavirus, for *Cryptosporidium* species there is no vaccine and the only FDA-approved therapy—nitazoxanide—is ineffective in malnourished children and immunocompromised individuals (8–11).

## The promise of vaccination

**Figure 1:**
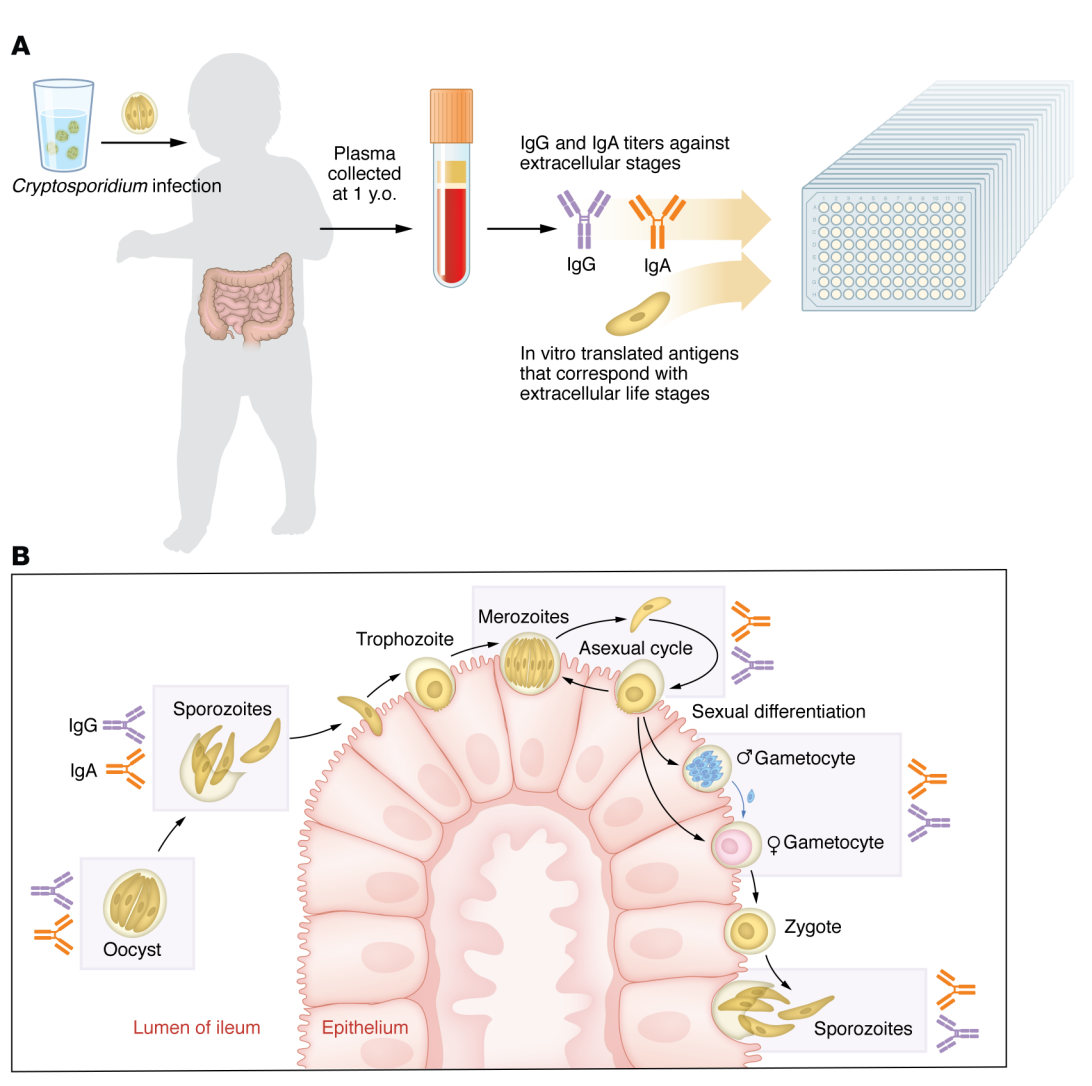
Children produce antibodies against *Cryptosporidium* proteins specific to the extracellular stages of the parasite’s lifecycle. (**A**) Gilchrist and Campo, et al. (18) profiled antibody responses in children previously infected by *Cryptosporidium*. The authors took serum from children at one year old and measured IgG and IgA against an array of proteins with signal peptide and/or transmembrane domains that correspond with Cryptosporidium extracellular parasite stages: gametocyte, sporozoite, merozoite, oocyst. (**B**) *Cryptosporidium* infection occurs after ingestion of infectious oocysts in contaminated water. Oocysts hatch in the small intestinal lumen to release four motile sporozoites that invade the apical side of intestinal epithelial cells (IECs). After invasion, sporozoites develop into intracellular trophozoites, which divide into eight merozoites that can exit the host cell and invade neighboring cells to establish a new infection. Merozoites can also develop into a trophozoite in new cells, thus undergoing asexual replication. After three rounds of asexual replication, parasites develop into male or female forms. Males exit the host cell and fertilization of a female gamete in another cell in the same host results in the formation of a zygote that can divide into four sporozoites to establish a new oocyst. New oocysts released in the feces can infect new hosts or hatch within the same host to autoinfect. Antibodies against seven proteins, expressed during the extracellular stages of *Cryptosporidium*’s lifecycle, were associated with protection from reinfection.

For *Cryptosporidium*, there are good experimental and epidemiological data for protective immune memory: human challenge studies showed that volunteers with prior cryptosporidiosis required a higher dose of oocysts for infection, and during natural infection, subsequent *Cryptosporidium* exposure resulted in milder symptoms (12–14). Based on these observations, it should be feasible to develop a vaccine to prevent or limit *Cryptosporidium* infection, which could substantially reduce child morbidity and mortality (15). Development of a vaccine requires knowledge of proteins made by the parasite that are immunogenic and associated with protection. One challenge with *Cryptosporidium* involves the complex life cycle of this particular eukaryotic parasite; it cycles through several intracellular and extracellular invasive stages and releases infectious oocysts into the environment (Figure 1) (16). Each life stage expresses some shared and unique proteins, and the potential for these factors to elicit an immune response remains largely unexplored (17). In addition, there are several species of *Cryptosporidium* that infect humans (*C*. *hominis, C*. *parvum,* and *C*. *meleagridis*), which adds to the diversity of potential immunogenic proteins. In this issue of the *JCI*, Gilchrist, Campo, and coauthors investigated whether children infected by *Cryptosporidium* developed antibodies against parasite proteins (18). This Bangladeshi cohort involved infants longitudinally followed for infection from birth to three years of age, where, by the end of the first year, 27.5% of infants had been infected at least once, and most children were reinfected by age three. Gilchrist and Campo, et al. took sera from one-year old participants to profile for antibodies against *Cryptosporidium* and then tracked reinfection in years two and three of life. The authors focused on antibodies against proteins from the extracellular life stages of the parasite, hypothesizing that these stage-specific antibodies may more effectively prevent infection compared with antibodies that targeted intracellular stages (Figure 1). They identified 233 *Cryptosporidium*-derived proteins that elicited serum antibodies. Of these 233 proteins, antibodies against seven (Cp23, Cp17, Gp600, CpMuc8, CpSMP1, CpCCDC, and CpCorA) were associated with protection from reinfection (18). Three of these (Cp23, Cp17, and Gp600) are current vaccine candidates. The other four proteins (CpMuc8, CpSMP1, and CpCCDC) expand the list of candidates to include in a potential vaccine.

## B cells and T cells in protection from *Cryptosporidium*

Gilchrist and Campo, et al. focused on antibodies as correlates of protection against *Cryptosporidium* and made several important conclusions regarding humoral responses. First, the authors noted that protection was not associated with antibody breadth, i.e. the number of unique antigens recognized by a given child. This finding was of interest, given that previously infected children had greater antibody breadth compared with children without documented infection during their first year of life. In addition, *Cryptosporidium* proteins recognized by each child tended to be unique, with each antigen recognized by only a subset of individuals, a result that suggests a paucity of immunodominant antigens. Both of these features may reflect the large number of potential epitopes encoded by *Cryptosporidium*—which, as a eukaryote, contains a larger genome than viruses or bacteria (approximately nine million base pairs for *Cryptosporidium* compared to four million for *E*. *coli* and less than one million for viruses). Furthermore, Gilchrist, Campo, and authors found that antibodies recognizing the polymorphic Gp40 protein, which is used to genotype *Cryptosporidium hominis* due to domains within the protein that vary across strains, were specific to the genotype of infecting *Cryptosporidium*. Thus, differences between species and strains of *Cryptosporidium* and their ability to sexually recombine would expand the range of antigenic targets a host is exposed to, and, consequently, repeated infections may increase the breadth of antibody responses. However, breadth on its own does not appear to increase protection, but rather harboring antibodies against a specific subset of antigens identified by Gilchrist and Campo, et al. promotes protection (18).

One reason to focus on serum antibodies is that they are relatively easy to measure and are already an established correlate of protection for other vaccines used in humans. However, mouse models have shown that B cell responses are dispensable for protection against primary infection by *Cryptosporidium*, perhaps because primary infections tend to resolve in less than 14 days, before the induction of a robust germinal center response (19, 20). It also seems likely that the production of mucosal antibodies will be the most relevant to *Cryptosporidum* biology. While mucosal antibodies were not investigated in the current study, they have previously been shown to correlate with protection against reinfection and malnutrition (21). Thus, these and prior studies indicate that antibodies are important for protection against reinfection (18).

Besides B cells, mucosal effector T cells (CD4^+^ T cells in particular) are thought to provide a major adaptive immune component that protects against *Cryptosporidium*. This conclusion is based on mouse models and findings related to primary and acquired immune deficiencies that associate with severe infection characterized by defects in T cells (20, 22). It is possible that the correlation of antibodies with protection reflects a readout of CD4^+^ T cell functions, which are required to generate high-affinity and class-switched antibodies. Thus, targeting T cells for vaccination will also be important for the induction of T cells that also support robust B cell responses. Consequently, it seems that there are two main goals for vaccination against *Cryptosporidium*: (a) vaccination must induce T and B cell responses and (b) these responses should include mucosal tissue. Live-attenuated vaccines are often utilized to induce mucosal antibody responses, as is the case with the oral poliovirus and rotavirus vaccines (23, 24). However, both mucosal vaccines are centered around eliciting neutralizing antibodies rather than T cell responses. For *Cryptosporidium*, attenuated vaccines are less feasible because the parasite can sexually recombine within a single host, which has the potential to allow live vaccines to lose attenuation through recombination with parasites in the wild. mRNA vaccination has been shown to induce robust systemic T cell responses to SARS-CoV2, and approaches to combine mRNA vaccination with intranasal boosting are being investigated to induce respiratory tissue–resident memory T cells (25, 26). A similar approach may be relevant to induce mucosal responses to *Cryptosporidium* without the risks of live-attenuated vaccines. Identifying the targets of protective T cell responses for inclusion in an mRNA vaccine will be important. Cp23 has already been shown to stimulate cellular responses in mice, and the other candidates identified by Gilchrist and Campo, et al. may hold promise as targets of cellular immunity (27). Nevertheless, considerable challenges remain to establish procedures that induce long-lived, local, parasite-specific antibody responses while also eliciting parasite-clearing T cell responses.

## Version 1 08/15/2023

Electronic publication
